# Serum cystatin is a useful marker for the diagnosis of acute kidney injury in critically ill children: prospective cohort study

**DOI:** 10.1186/s12882-016-0346-z

**Published:** 2016-09-13

**Authors:** Osama Y. Safdar, Mohammed Shalaby, Norah Khathlan, Bassem Elattal, Mohammed Bin Joubah, Esraa Bukahri, Mafaza Saber, Arwa Alahadal, Hala Aljariry, Safaa Gasim, Afnan Hadadi, Abdullah Alqahtani, Roaa Awleyakhan, Jameela A. Kari

**Affiliations:** 1 Pediatric Nephrology Unit, Department of Pediatrics, King Abdulaziz University, Jeddah, Kingdom of Saudi Arabia; 2 Intensive Care Unit, Department of Pediatrics, King Abdulaziz University, Jeddah, Kingdom of Saudi Arabia; 3 Department of Pediatrics, King Abdulaziz University, Jeddah, Kingdom of Saudi Arabia; 4 Faculty of Medicine, King Abdulaziz University, Jeddah, Saudi Arabia; 5 King Abdullah Specialized Children Hospital, King Abdulaziz Medical City, Riyadh, Kingdom of Saudi Arabia; 6 College of Medicine, King Abdulaziz University, Jeddah, Kingdom of Saudi Arabia; 7 Pediatric Department, King Abdulaziz University Hospital, P.O. Box 14071, Alsulimania, Jeddah, 21414 Kingdom of Saudi Arabia

**Keywords:** Pediatric, Acute kidney injury, Cystatin C, Creatinine

## Abstract

**Background:**

Acute kidney injury (AKI) has been associated with high morbidity and mortality rates among critically ill children. Cystatin C is a protease inhibitor, and studies have shown that it is a promising marker for the early diagnosis of AKI. Our goal in this study was to assess whether serum cystatin C could serve as an accurate marker for the diagnosis of AKI.

**Methods:**

This prospective study was undertaken in the pediatric intensive care unit at King Abdulaziz University Hospital. Serum creatinine and serum cystatin C levels were both measured in patients on admission (0 h) and at 6, 12, and 24 h after admission. AKI was diagnosed according to the modified pRIFLE criteria. Receiver operating characteristic (ROC) curve analysis was performed to assess the utility of serum cystatin C for diagnosing AKI.

**Results:**

A total of 62 patients were enrolled in this study, and 32 were diagnosed with AKI according to the modified pRIFLE criteria (51.4 %). The area under the ROC curve for serum cystatin indicated that it was a good marker for the diagnosis of AKI at 0, 6, 12 and 24 h, with sensitivities of 78, 94, 94 and 83 %, respectively. However, the specificities of serum cystatin C at 0, 6, 12, and 24 h were 57, 57, 60 and 50 %, respectively. The optimal cutoff value was 0.645 mg/L. The area under the ROC for serum creatinine showed sensitivities of 50, 65.4, 69.2 and 57.7 % and specificities of 67.7, 70, 60 and 70 % at 0, 6, 12 and 24 h, respectively. The optimal cutoff value for serum creatinine was 30 μmol/l. Comparisons of ROC curves revealed that serum cystatin C was superior to serum creatinine for the diagnosis of AKI at 12 h (*p* = 0.03), but no differences were detected at 0, 6 or 24 h.

**Conclusion:**

Serum cystatin is a sensitive, but not a specific, marker for the diagnosis of AKI in critically ill children.

## Background

Acute kidney injury (AKI) is common among pediatric patients who are admitted to intensive care units (ICUs)/critical care units, and this condition has been associated with high morbidity and mortality rates [1, 2]. The etiology of AKI varies from simple dehydration, which is managed by volume repletion, to multifactorial causes in critically ill children, and it can require renal replacement therapy. Various clinical criteria have emerged with the goal of diagnosing AKI and classifying it along a spectrum according to severity, including the RIFLE [3], AKI Network (AKIN) [4], and Kidney Disease Improving Global Outcomes (KDIGO) criteria [5]. All of these criteria depend heavily on the serum creatinine level and/or urine output (UOP).

However, the use of the serum creatinine level has many drawbacks, including variability according to age and sex and dependence on muscle mass, making it unsuitable for diagnosis of malnourished children. There is also a delay between the occurrence of significant renal damage and the increase in the serum creatinine level. Further, it is difficult to rely solely on measuring UOP for AKI diagnosis because nephrotoxic medications and interstitial kidney disease are associated with normal to increased UOP. Many biological markers have emerged with the goal of detecting AKI early, including cystatin C [6], neutrophil gelatinase-associated lipocalin [7], kidney injury molecule-1 [8], and interleukin-18 [9].

Cystatin C is 13-kDa cysteine protease inhibitor that is produced by all nucleated cells at a constant rate. This compound is freely filtered by the glomeruli and completely catabolized by the proximal tubules with no secretion; thus, it is promising for use in glomerular filtration rate (GFR) estimation [10, 11].

In pediatric clinical studies, cystatin C has shown a high predictive value for diagnosis, and some studies have even shown that it is superior to serum creatinine in the early detection of AKI. Recently, cystatin C has been shown to be a sensitive marker for early AKI diagnosis in children admitted to a pediatric intensive care unit (PICU) [12]. Serum and urinary cystatin C have also been shown to be good markers for AKI in heterogeneous pediatric populations, including patients from an emergency department [13], a neonatal ICU [14], and a post-cardiac surgery unit [15].

In this study, we aimed to assess the utility of serum cystatin C for AKI diagnosis in children admitted to a PICU.

## Methods

This was a prospective study of children admitted to the PICU at King Abdulaziz University Hospital, Jeddah, Saudi Arabia from June to August, 2013.

Any sick patient ranging in age from over 1 month to 18 years of age who was admitted to the PICU was considered at risk of developing AKI and eligible for inclusion in the study. They were screened for AKI by performing serial measurements of serum creatinine levels during the first 48 h after admission to assess changes in the estimated GFR as well as in the serum cystatin C level. The exclusion criteria included the following: patients who were known to have chronic kidney disease (CKD) at stage 3 to 5 (GFR < 60 mL/1.73 m^2^/min); and patients who were stable clinically and were admitted only for elective procedures, such as central line insertion. Neonates were not included because they are not allowed to be admitted to the PICU as per hospital regulations.

### Data collection

We collected demographic data, medical histories, reasons for admission to the PICU, diagnoses, fluid balance information, anthropometric measurements, laboratory test results, and data on the need for renal replacement therapy.

The modified pRIFLE criteria were used to diagnose AKI according to the serum creatinine level and UOP at the time of admission or during treatment at the PICU [16]. The serum creatinine and serum cystatin C levels were measured at the time of admission (zero time) and at 6, 12 and 24 h after admission. Approximately 5 mL of blood was obtained each time.

### Laboratory tests

The serum creatinine level was measured using the Jaffe kinetic spectrophotometric method, which is known for its low specificity because many chromogenic substances interfere with the accurate determination of the GFR and calibration is difficult. The serum cystatin C level was measured according to the manufacturer’s protocol supplied with the ELISA kit, and the reference interval was calculated nonparametrically and was determined to be 0.53–0.95 mg/L. This range of values pertains to the central 95 % of the population. In ELISA, turbidity and particles in samples may interfere with measurements. Therefore, samples containing particles were centrifuged prior to performing this assay.

### AKI analysis

The AKI patients were classified into three categories (Risk, Injury, Failure) based on the magnitude of change in the estimated GFR or in UOP as follows: risk stage, 25 % decrease in the GFR and UOP < 0.5 mL/kg/h for 8 h; injury stage, 50 % decrease in the GFR and/or UOP < 0.5 mL/kg/h after 16 h; and failure stage, 75 % decrease in the GFR or GFR < 35 mL/min/1.73 m^2^ and/or UOP < 0.3 mL/kg/h for 24 h or anuria for 12 h [16]. The baseline creatinine level was defined as the last creatinine measurement within the previous 6 months prior to PICU admission; for those patients who were admitted for the first time with no previous creatinine measurement, we used the average normal GFR according to the age and sex of the child [17]. The estimated glomerular filtration rate (eGFR) was calculated using the bedside Schwartz formula [18], which utilizes the concentration of serum creatinine (μmol/l), the height of the child (cm) and a constant to estimate the glomerular filtration rate:

$$ \mathrm{eGFR}=\frac{k\times Height}{Serum\kern0.5em  Creatinine} $$

Where *k* is a constant that depends on muscle mass, which itself varies with a child’s age: For infants and children aged 1 to 12 years, K = 0.55. For children above 12 years old, K for girls 0.55 and for boys 0.70 [18].

### Statistical analysis

The following statistical analyses were performed using IBM SPSS statistics version 20.0:

1. The Shapiro-Wilk test was used to test the normality of the study sample.
2. Descriptive statistics were used to calculate the mean, standard deviation, median, quartiles.
3. The Chi-square test for association was used to determine whether there was any relationship between the categorical variables and AKI status.
4. Receiver operating characteristic (ROC) curves were constructed to provide a natural common scale for comparing different predictors that were measured in different units and to interpret sensitivity and specificity levels to determine the best cutoff value. The Hanley method was used to compare the ROC curves of serum cystatin C and serum creatinine.
5. The Mann-Whitney test was used to compare data that were non-normally distributed.

## Results

### Demographic characteristics

A total of 62 patients, comprising 35 boys and 27 girls, were recruited for the study.

The median age for AKI occurrence was 18 months, with an interquartile range of 8 to 42 months, while for non-AKI patients, the median age was 20.5 months, with an interquartile range of 6 to 54 months. Age and gender had no effect on the development of AKI. According to the modified pRIFLE criteria, 32 patients had AKI (51.6 %), and they were categorized as follows: Risk class, 18 patients (56.3 %); Injury class, 10 patients (31.5 %); and Failure class, 4 patients (12.5) based on modified pRIFLE criteria.

The most common cause of AKI was sepsis, which occurred in 16 patients (50 %), followed by hypoxia, defined as prolonged low oxygen saturation related to respiratory or cardiac disease, which was found in 13 patients (39 %). According to the pRIFLE criteria, only 4 patients were diagnosed with AKI at the time of admission (0 time), 10 patients at 6 h, 6 patients at 12 h and 12 patients at 24 h.

A true baseline creatinine level was available for 28 patients (14 with AKI and 14 without AKI; measured during the previous 6 months), and for the remaining 34 patients (18 with AKI and 16 without AKI), the GFR was estimated using the Schwartz formula [17].

The Mann-Whitney test showed that baseline GFR was not different between the non-AKI and AKI groups The non-AKI mean rank was 32.87, and the AKI mean rank was 30.22 (see Table 1).

**Table 1:** Demographic data for both patient groups: acute kidney injury (AKI) and non-acute kidney injury (non-AKI)

Variables No. of patients	AKI 32 patients	Non-AKI 30 patients	*P*-value
Age (months)	18 (8–42)	20.5 (6–54)	0.05
Sex			
Male	19 (59.3 %)	16 (53.3 %)	0.79
Female	13 (40.7 %)	14 (46.7 %)	
RIFLE stage for AKI group			
RIFLE	18 (56.3 %)		
Injury	10 (31.25 %)		
Failure	4 (12.5 %)		
Possible etiologies for AKI group (*N* = 32)			
Hypoxia/ischemia/ATN	13 (40.6 %)		
Sepsis	16 (50.0 %)		
Glomerulonephritis	2 (6.3 %)		
Urinary tract obstruction	1 (3.1 %)		
Nephrology consultation	13 (40.6 %)		
Diuretic use	23 (71.8 %)		
Renal replacement	1 (3.1 %)		
Basal GFR (ml/min/1.73 m^2^) (median-interquartile range)	80 (66–96)	109 (79–124)	0.08
Basal creatinine (μmol/l) (median-interquartile range)	41.5 (31–51.2)	29.4 (24–34,4)	0.05
Basal cystatin C (mg/l) (median-interquartile range)	0.901.7 (0.802.5–1.502)	0.611.6 (0.549–0.672)	0.05
Mortality	4 (12.5 %)	1 (3.3 %)	0.114
Creatinine on discharge (μmol/l) (median-interquartile range)	28.0 (23–35)	28.72 (24–34.2)	0.832
GFR at discharge (ml/min/1.73 m^2^) (median-interquartile range)	102.5 (82.5–114.5)	104.7 (84–120)	0.535

The estimated GFR on discharge was higher in the non-AKI comparing to AKI groups The non-AKI mean rank was 32.69, and the AKI mean rank was 24.00 (see Table 1).

### Utility of serum cystatin C for diagnosing AKI

We performed ROC analysis at each time point to assess the utility of serum cystatin C for diagnosing AKI. An investigational analysis revealed that the best cutoff values for serum cystatin C and serum creatinine were 0.645 mg/L and 30 μmol/l, respectively.

**Fig. 1:**
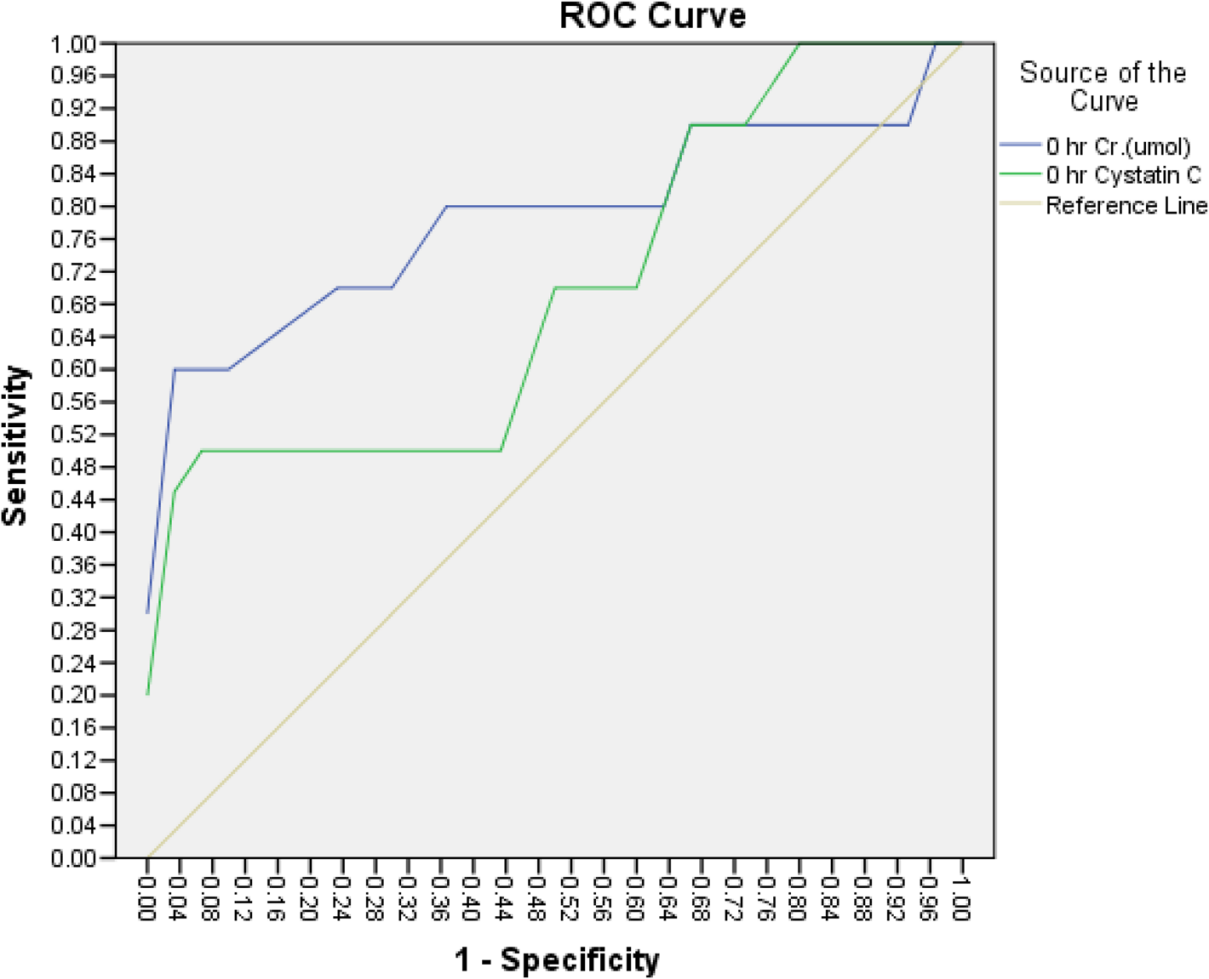
ROC analysis of cysatatin C at 0 h for the diagnosis of AKI had a sensitivity of 78 % and a specificity of 57 % with a cutoff 0.645, while serum creatinine had a sensitivity of 50 % and a specificity of 67.7 % with a cutoff value of 30 umol/l

At 0 h, ROC curve analysis revealed that the area under the curve (AUC) was 0.825 (95 % CI: 0.694–0.956), with a sensitivity of 78 % and a specificity of 57 %. In comparison, serum creatinine at 0 h had a sensitivity of 50 % and a specificity of 67.7 %, with an AUC of 0.733 (95 % CI: 0.605–0.867). The ROC curves were not significantly different (*p* = 0.26; see Fig. 1).

**Fig. 2:**
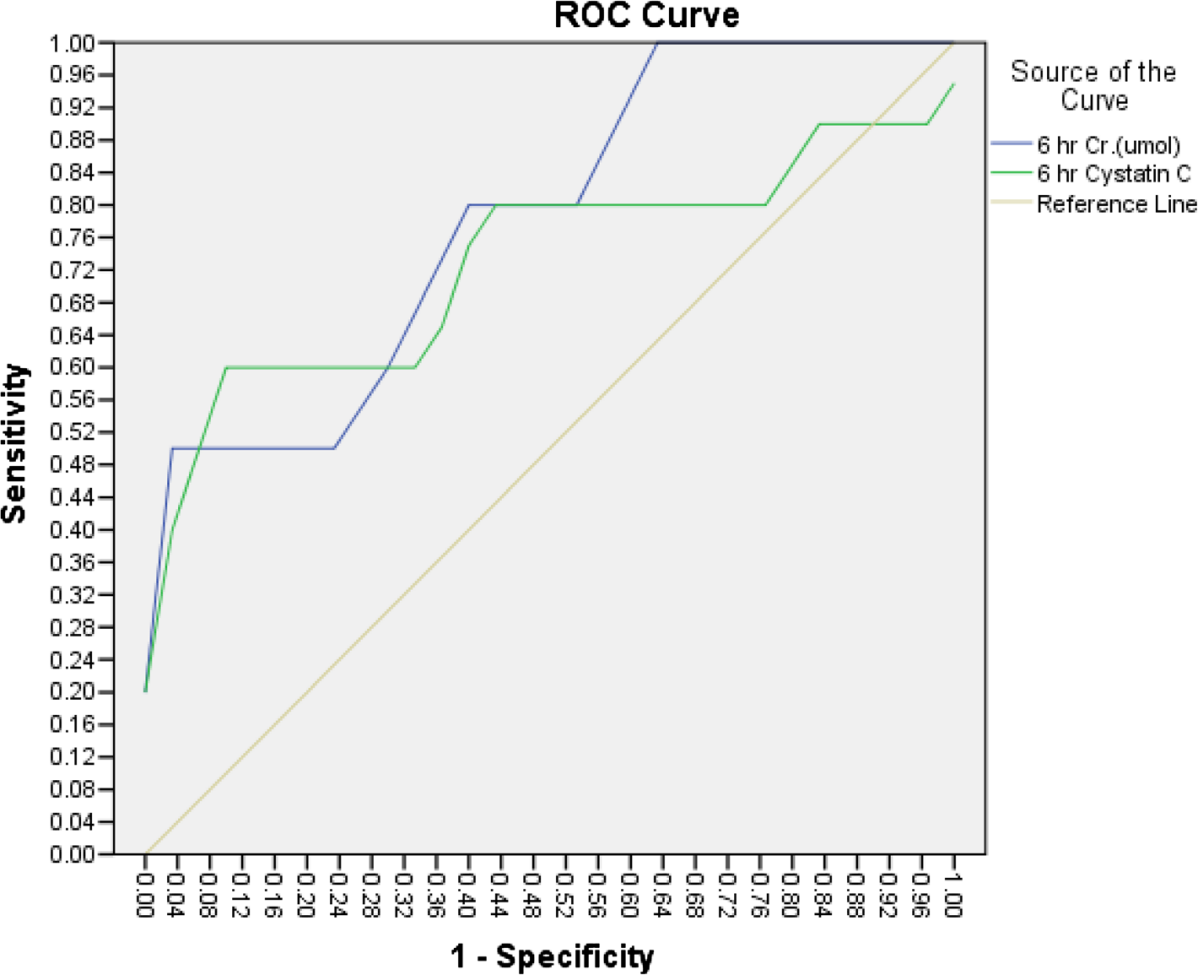
At 6 h, ROC analysis of serum cystatin C revealed a sensitivity of 94 % and a specificity of 57 % with cut-off value of 0.645 mg/l, while ROC analysis of serum creatinine showed a sensitivity of 65.4 % and a specificity of 70 % with cut-off value of 30 umol/l. This difference was not significant (*p* = 0.15)

At 6 h, the AUC for serum cystatin C was 0.825 (95 % CI: 0.694–0.956), with a sensitivity of 94 % and a specificity of 57 %. In comparison, the area under the curve for serum creatinine was 0.704, with a sensitivity of 65.4 % and a specificity of 70 % (95 % CI: 0.567–0.841). This difference was not significant (*p* = 0.15; see Fig. 2).

**Fig. 3:**
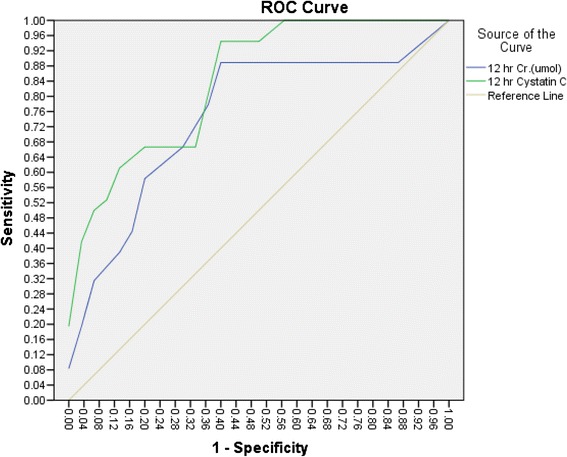
At 12 h, ROC analysis of serum cystatin C revealed a sensitivity of 94 % and a specificity of 60 % with cut-off value of 0.645 mg/l, while ROC analysis of serum creatinine showed a sensitivity of 69.2 % and a specificity of 60 % with cut-off value of 30 umol/l. This difference was significant (*p* = 0.03)

At 12 h, the AUC for serum cystatin C was 0.843 (95 % CI: 0.732–0.953), with a sensitivity of 94 % and a specificity of 60 %. In comparison, the AUC for serum creatinine was 0.658 (95 % CI: 0.510–0.805), with a sensitivity of 69.2 % and a specificity of 60 %. This difference was significant (*p* = 0.03; see Fig. 3).

**Fig. 4:**
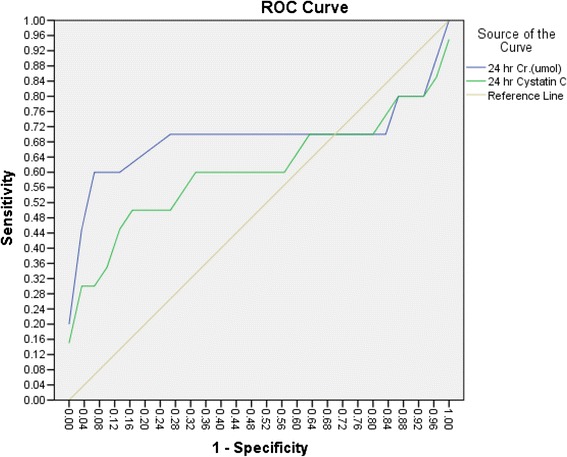
At 24 h, ROC analysis of serum cystatin C revealed a sensitivity of 83 % and a specificity of 50 % with cut-off value of 0.645 mg/l, while ROC analysis of serum creatinine showed a sensitivity of 57.7 % and a specificity of 70 % with cut-off value of 30 umol/l. This difference was not significant (*p* = 0.18)

Finally, at 24 h, the AUC for serum cystatin C was 0.780 (95 % CI: 0.634–0.925), with a sensitivity of 83 % and a specificity of 50 %. In contrast, the AUC for serum creatinine was 0.658 (95 % CI: 0.504–0.812), with a sensitivity of 57.7 % and a specificity of 70 %. This difference was not significant (*p* = 0.18; see Fig. 4).

We conclude that serum cystatin C has a good sensitivity but lacks specificity for the diagnosis of AKI during the first 24 h after ICU admission. The diagnostic ability of serum cystatin C was superior to that of serum creatinine at 12 h, but no differences were detected at 0 h, 24 h or 48 h.

## Discussion

In our cohort, 51.6 % of the admitted patients were diagnosed with AKI. This incidence is similar to those observed in other pediatric cohorts [2, 15], indicating that AKI is a significant and common issue among critically ill children.

The mortality rate is high among children admitted to ICUs who are diagnosed with AKI, with reported rates ranging from 40 to 46 % [19–21]. A study conducted in Poland showed that AKI is associated with a 4.4 times higher risk of death compared with the total mortality rate for PICU patients [22]. Another study conducted in Canada has reported that AKI is an independent risk factor for mortality, a longer length of hospital stay and prolonged mechanical ventilation in critically ill children [23]. Similarly, we have reported that hypervolemia, mechanical ventilation, RIFLE class failure and renal replacement therapy initiation are associated with a higher likelihood of death for AKI patients in a PICU [20].

We used only the pRIFLE definition of AKI in this study and did not consider the other definitions of AKIN and KDIGO because Sutherland SM et al. have recently shown that all three definitions are equally excellent and provide good discrimination in children [24].

We found that early measurement of the serum cystatin C level within first 24 h is a sensitive predictor of AKI in critically ill children. This finding is similar to those of other cohort studies of children admitted to ICUs. Herrero-Morin et al. have shown that the serum cystatin C and beta-2 microglobulin levels are more strongly correlated with creatinine clearance than with serum creatinine in children with AKI admitted to an ICU [25]. Another study conducted in Iran has reported that the sensitivity and specificity of serum cystatin C for diagnosing AKI are 73.9 % and 78.9, respectively, using 0.6 mg/ml as the cutoff value [12]. Similarly, a study conducted by Lagos-Arevaldo et al. of 160 non-cardiac patients admitted to a PICU has reported that serum cystatin C has more diagnostic accuracy than serum creatinine and a greater predictive value for clinical outcomes [26].

Serum cystatin C has also been reported to have a higher predictive value for early AKI diagnosis in children who have undergone cardiac surgery. In a study of 100 children who underwent cardiopulmonary bypass, Hassinger et al. showed that serum cystatin C is highly sensitive and predictive in diagnosing AKI, with an AUC of the ROC curve of 0.834–0.875 [27]. Similarly, Krawczeski et al. conducted a study of 347 children showing that serum cystatin C is an early predictive biomarker for AKI and clinical outcome after pediatric cardiopulmonary bypass [15].

In our cohort, we did not include the neonatal age group, which could have biased our results. As previously stated, we did not include neonates because the hospital policy does not allow for admission of babies <28 days of age to the PICU. However, serum cystatin C has been demonstrated to be sensitive marker for AKI diagnosis in asphyxiated babies [14].

Other studies have shown contradictory results and have found that cystatin C is a poor marker for AKI diagnosis. A recent study of 32 pediatric patients admitted to an ICU demonstrated that the use of serum cystatin C is not superior to that of serum creatinine for the diagnosis of AKI [28]. This finding is similar to that of a clinical study performed by Royakkers et al., who demonstrated that both serum and urine cystatin C are poor markers for the diagnosis of AKI and that they do not predict the need for renal replacement therapy [29].

There is no clear explanation for these conflicting results, but they might reflect the heterogeneity of the populations assessed and differences in the AKI definitions used; another possibility is that different cutoff values for serum creatinine were used to diagnose AKI in these studies.

We found that serum cystatin C lacked specificity for the diagnosis of AKI. This finding could be explained by the observation that the serum cystatin C level can be affected by factors such as hyper/hypothyroidism [30], steroid treatment [31], growth hormone [32], and insulin [33].

Despite the utility of serum cystatin C for the early diagnosis of AKI, it is not clear whether it has any impact on or role in the management of this condition. AKI management currently involves supportive management and renal replacement therapy if needed. With the exception of immunosuppressive medication administration to patients with an immune-mediated disease, no pharmaceutical management strategy has been found to improve the outcome of AKI. In a randomized controlled trial, Ricci et al. showed that fenoldopam infusion in children with congenital heart disease who were undergoing cardiopulmonary bypass decreased the levels of urinary cystatin C and NGAL at the end of surgery and at 12 h after surgery [34]. The incidence of AKI was lower in the fenoldopam group (50 %) compared with the placebo group (72 %); however, this difference was not significant. Thus, further prospective studies are required to establish a link between cystatin C and interventional therapy that can prevent or halt the progression of AKI and to assess the role of cystatin C in improving patient outcomes.

Another potential source of bias is that we only included patients who had been admitted to the PICU and did not include those who came to the emergency department and are at risk of community-acquired AKI. However serum cystatin C has been reported to be a good marker for AKI diagnosis in patients presenting at an emergency department [13].

The tests for both the serum creatinine and serum cystatin C levels are automated, and the labor costs are minimal. However, the cost of measuring the serum cystatin C level is more than that of measuring the serum creatinine level because of the higher cost of reagents. The cost of cystatin C reagents is $4 per test, which is 20 times the cost of the creatinine test using the Jaffé reaction ($0.20) and about 3 times the cost of enzymatic creatinine assay. However, this extra cost of the cystatin C test should not preclude its use [35].

## Conclusions

To our knowledge, this is the first study conducted in Saudi Arabia to assess the utility of serum cystatin C for the early diagnosis of AKI. Our study has several limitations, including the following: 1) it was a single-center study with a relatively small number of patients; 2) the definition of AKI used in this study was based on changes in the eGFR according to the Schwartz formula and using serum creatinine, which has several drawbacks, as previously mentioned, and might have compromised the accuracy of serum cystatin C for the detection of AKI. 3) We lack data regarding other factors that could affect levels of serum cystatin C, such as steroid therapy, the use of insulin and thyroid function test results.

We believe that further prospective studies are needed with evaluation of several multicenter studies, which will increase the sample size. In addition, a prolonged follow-up period is needed to assess the long-term outcomes of AKI and to evaluate the possible role of cystatin in the prediction of long-term outcome.

AKI is common in critically ill children.

Serum cystatin C is a sensitive marker for diagnosis of AKI in critically ill children if it is measured within the first 24 h of admission.

Serum cystatin C lacks specificity for AKI diagnosis in patients admitted to the PICU.

## Abbreviations

AKI: Acute kidney injury
AKIN: AKI network
AUC: Area under the curve
CKD: Chronic kidney disease
GFR: Glomerular filtration rate
ICU: Intensive care unit
KDIGO: Kidney disease improving global outcome
PICU: Pediatric intensive care unit
ROC: Receiver operating characteristic
UOP: Urine output
